# Eight ‘truths’ about suicide

**DOI:** 10.1192/bjb.2023.75

**Published:** 2024-12

**Authors:** Rachel Gibbons

**Affiliations:** Royal College of Psychiatrists, London, UK

**Keywords:** Suicide, psychodynamics, post-traumatic stress disorder, self-harm, trauma and stressor-related disorders

## Abstract

This paper summarises themes that have emerged from 14 years of study of suicide and work with those bereaved. It is based on a talk given in many clinical settings over the past 10 years. I describe my own emotional journey following impactful deaths and summarise personal ‘truths’ about suicide that have emerged over time. Case studies used for illustration are composites taken from clinical practice; accounts of relatives and other survivors of suicide; and data taken from many sources including suicide audits in mental health organisations, the police and transport services, and from the examination of coroners’ records. The intention is to assist open dialogue about the nature of suicide, to contribute to the understanding of the impact on those bereaved and to encourage open-hearted clinical engagement with those who are suicidal.

*Every surgeon carries within himself a small cemetery, where from time to time he goes to pray*

Rene Leriche^1^

Suicide has defined my psychiatric career. I was delighted to get my first consultant job working in an in-patient unit. However, in my second week I had my first patient die by suicide. In my third week I had my second patient die by suicide. In my third month there was a very distressing and violent death by suicide on the ward. Shockingly, this latter death was initially treated by the police as a murder. I know now that this is not uncommon.^2^

After these tragic deaths, the part of me that thought I could bring something therapeutic to my work as a consultant psychiatrist died. How could I have had three deaths like this? It must have something to do with me. I must have caused or at the very least significantly contributed to them. Between each death, I had no time to reinstate my defences, and after my first 6 months as a consultant I was permeated by the experience of suicide. At the time I felt deeply ashamed, humiliated and alone. In common with many doctors, I continued working.^3^ I was profoundly divided. One part of me remained in the doctor role, kept the show on the road, appeared at work each day and went through the motions. The other part was tucked away inside, in a state of abject panic, terrified by every patient that I had contact with, incapacitated and unable to make even the simplest clinical decision. In retrospect, I now know that I had, and have, a post-traumatic stress disorder, and many other clinicians are also suffering in this way.^3,4^

In 2009, no one around me was talking about suicide; there was no support and no resources to normalise my experience. It was at this time that I met the chaplain of Beachy Head who offered me his thoughts from 20 years of experience. He said that ‘to help a truly suicidal person you have to approach them with an open heart’. The concept of a compassionate and undefended ‘open-hearted’ engagement with someone in suicidal pain felt an impossible goal at that time. My internal world was shattered and full of dread, and the mental health services I worked within were dominated by the fear of patients, perceived as carriers of ‘risk’ and defended against by risk assessments.

I discovered two colleagues in very similar positions and comparable psychological states. We formed a confidential peer support group where we could be vulnerable. We met monthly and talked about these deaths and our responses to them. Gradually, over time, I started feeling that I could breathe again. This peer support group is still running 14 years later, and we have heard over 200 cases of suicide.^5^ This group is now being developed in other mental health environments. There is one held by the Royal College of Psychiatrists linked with the Psychiatrists Support Service.

## The philosophical question of suicide

*There is but one truly serious philosophical problem and that is suicide.*

Camus^6^

After hearing many stories of deaths by suicide and of clinicians’ emotional responses to them, patterns or ‘truths’ started to appear, and awareness emerged that what we were discovering about suicide did not correspond with what we were being told in the wider social environment. Some of these ‘truths’ may seem obvious; however, the obvious can be lost in the anxiety that overwhelms individuals and systems after a death. The case examples used are composites of cases taken from clinical practice, those recounted by relatives and other survivors of suicide, and data from many sources including audits in mental health organisations, the police and transport services, and the examination of coroners’ records. All terms marked by an asterisk are defined in Table 1.

**Table 1:** Definitions of terms

Term	Definition
The unconscious	Freud divided the mind into the conscious and the unconscious. The unconscious holds socially unacceptable ideas, anxiety-producing wishes or desires, traumatic memories and painful emotions. What was once a controversial idea is now widely accepted and may account for over 90% of the brain's functioning.^37^
Unconscious mental mechanisms	Information processing of the material in the unconscious.
Split in the ego	A division in the sense of self, where two parts of the self function side by side without influencing each other, one taking reality into account and the other disavowing reality; each side not communicating with the other.
Ego	One of three parts of Freud's ‘structural model’ of the psyche that mediates between the ‘id’ and the ‘superego’. The ego engages with reality and it is where self-identity is located. The id is entirely unconscious and holds the basic instinctual urges, such as aggression and sexuality. The superego functions as a conscience, repressing what it considers to be morally unacceptable.
Object	In object relations theory, early infantile experience is described as relationally based. The attachment energy, or libido, is focused on people and things, which are defined as ‘objects’. There is an internalisation of representations of these ‘objects’ that populate the mind and increase in complexity and depth through mourning and separation.
Projection	An unconscious defence to expel unacceptable aspects of the self into the outside world; these are then experienced as belonging to someone or something else.
Unconscious defence mechanisms	Socially unacceptable ideas, anxiety-producing wishes or desires, traumatic memories and painful emotions are held outside conscious awareness by unconscious defence mechanisms. These take many different forms.
Suicidal fantasy	Delusional conviction and distortion of reality where suicide allows for a new form of continued life. Campbell and Hale define five forms.^8^
Mentalise	The ability to reflect upon and understand your own state of mind; to have insight into what one is feeling, and why; to be able to conceptualise others’ mental states and recognise that they may be different to your own.
Symbolise	A symbol is an indirect form of representation that allows an individual to think about people, objects or events that are not concretely available. These symbols are available for utilisation intrapsychically to represent ideas, conflicts or wishes.
Location of disturbance	Psychological disturbance that is ‘located between people’ in the social area becomes diverted and placed into one person or group.^38^

If you have been affected by suicide yourself, please read this with caution.

## Eight ‘truths’ about suicide

### No. 1: suicide is not an accident

The causative factors in any individual suicide are complex and multifactorial. Some suicidologists believe that around 30–50% of the risk is genetic, and the aetiology then may start in the womb.^7^ Contemporary psychoanalysts such as Campbell, Hale and Maltsberger conceptualise suicide as resulting from complex unconscious* mental mechanisms* that we do not yet fully understand.^8,9^ In their view, the destructive pathway is set in motion as a response to loss where the capacity to mourn is overwhelmed. The failure in this process causes a split* within the personality structure (ego*), which allows the body to be treated as a separate object*, projected* into and experienced as the source of the pain which cannot be digested. In this state, the mind is perfused with ‘suicidal fantasies’, which are a ‘quasi-delusional psychic retreat’ from unbearable anxiety. The core belief of these fantasies is that liberation from the body will allow a ‘survival self’ to continue life in a pain-free ‘bodiless way’,^8^ allowing for an avoidance of the awareness of the finality of death.^10^ These fantasies can be seen on occasion in the symbolic nature of the suicidal acts. For example, people travel from all over the world to jump from the Golden Gate bridge in America (or Beachy Head in the UK), perhaps in a bid to transition through a portal or ‘Golden Gate’ to a pain-free heavenly world where their life can begin again.

*I really won't die, I'll just wake up and things will be different. That's how I feel tonight that I'm not really going to die, and the other one is, and I don't know how to explain that to you. I seem to be contradicting myself*

Patient^9^

### No. 2: we will never know why someone died by suicide

*The one thing families want to know is why. They ask me over and over again. ‘Why? Why? Why?’ I can only tell them that we do not know*.

Family Liaison Officer

The true reasons why someone has died by suicide will never be known. The person who could shed some light on the situation is no longer present, and even when people have been interviewed after making very serious attempts on their life, they are frequently unclear as to why they did it and can be as shocked and surprised as everyone else by their actions.^11,12^ Only around a quarter of people who die leave a suicide note, and they very rarely give any good reasons for their death.^13,14^ As a result, those bereaved are left in a state of unbearable pain and uncertainty. This can be temporarily mitigated by generating simple narratives where the death is conceptualised as an accident, or the result of their own or others’ negligence. These narratives allow the preservation of the memory of the person who is lost, and enable their role in their own death to be temporarily obscured.

*It was several months before I would have been able to think through my son's death … and then further years before I could place this in a perspective that allowed for the unknowability, the limits to intervention, and allowed agency of some kind to my son in his own death. There was a time when finding others responsible was important in preserving the integrity of my son …, finding causes in myself and others who did not keep him safe. It might help professionals to place the anger or blame they may experience from families in the context of the process of grief they (the families) are going through. The point is that families’ engagement with professionals in the early period may be shaped by their need for a narrative of the suicide with which they can live, driven by their need for emotional survival. Narratives of blame serve to protect a family's relationship with the one they love and have lost*.

Bereaved father^15^

### No. 3: the ‘act’ of suicide: suicide is an ‘acting out’ behaviour that results in death

*An act like this is prepared within the silence of the heart, as is a great work of art. The man himself is ignorant of it. One evening he pulls the trigger or jumps.*

Camus^6^

Campbell, Hale and Maltsberger describe suicide as an ‘acting out’ behaviour aimed at ridding the self of pain.^8,9^ ‘Acting out’ is an unconscious defence mechanism* where action takes the place of feeling. For many who make serious attempts on their life, the first sign that they feel in such overwhelming distress is that they act.^16^

*Case example: A young woman interviewed in ITU after a life-threatening overdose said that morning, she had said goodbye to her partner and they had discussed what they were going to have for dinner. At that time, she planned to see him that evening. When he left, she took an overdose. She was shocked and could offer no explanation. She said that some part of her must have been preparing to do this for a while*.

If the attempt is survived, the underlying distress can be recognised by the individual themselves and those around them. With this can come the development of concern and an opportunity for development and growth.^17^

### No. 4: everyone is shocked by the death

*Case example: A husband and wife, with no history of mental health problems, sit down to dinner and share a bottle of wine. They go to bed. The wife gets up in the morning and finds her husband has died by suicide in the bathroom during the night*.

There is much discussion about the predictability of individual suicide; however, many deaths are not preceded by warnings, and frequently those who die have hidden their intent from those around them.^18,19^ For the bereaved, it is important to recognise the death is a ‘bolt from the blue’ (a phrase often used by those bereaved) because very quickly narratives are constructed, as described above, where the death could have been foreseen, mistakes made and blame apportioned. The National Confidential Inquiry finds each year that around 73% of people who die by suicide have not been in touch with mental health services in the year preceding their death. For the 27% who have and have had a risk assessment tool filled in at their last contact, 83% have been rated as no or low risk.^20^ Even in the small group with recognised serious ongoing suicidality, there is often no expectation of the attempt at that point in time. Some are reported to appear to improve before their death.^21^

### No. 5: suicide appears to be either impulsive or premeditated

Deaths by suicide appear to be either impulsive or premeditated.^21^ There are numerous examples of apparently impulsive deaths, with many occurring in the middle of an argument or after a physical fight. In some cases, the suicidal act occurs within 5 min of the decision.^22,23^ Many deaths, however, have been clearly premeditated, often over long periods of time, without even the closest people to the deceased having any awareness of this preparation. In some cases, people can be very determined, secretive and inventive about how to end their lives.

*Case example: Mr P lived on his own and planned his death in meticulous detail. Over the previous months, he had rid himself of all his personal items, cleaned his flat, and left notes to family members and the coroner. Those close to him were very shocked to find evidence of this secret preparation because they had not noticed anything out of the ordinary in his behaviour. They said actually he had seemed to be in a better mood than he had for a long time*.

### No. 6: suicide is part of the human condition and is not just related to a mental health condition

The relationship between suicide and mental illness is complicated and poorly understood. Data indicates that a significant number of people do not have a diagnosed mental illness at the time of death, and some have no known history of mental distress.^24–29^ Whether this group has an underlying mental illness therefore cannot be established. The incapacity to mourn loss is thought to be an aetiological factor in both mental illness and suicide;^30^ however, the assumption of a direction of causality may be erroneous. Research shows that suicide follows antecedents that can be broadly categorised as life events including: significant physical illness, bereavement (particularly bereavement by suicide), relationship breakdown, being charged by the police (particularly with child sex offences) and domestic violence (for both perpetrator and victim).^24–29^

Thinkers about suicide such as Durkheim,^31^ Hillman,^32^ Sartre and Camus^6^ do not see mental illness as a primary cause of suicide. Durkheim considers suicide as a social phenomenon (egoistic, altruistic, anomic, fatalistic); Hillman regards to drive to suicide as a transformative impulse, as an attempt to go through the death experience with meaning, Sartre as an assertion of authentic human will in the face of absurdity, and Camus as a rational response to life's lack of meaning.

*Dying voluntarily implies that you have recognized even instinctively the ridiculous character of the habit, the absence of any profound reason for living, the insane character of that daily agitation, the uselessness of suffering.*

Camus^6^

If we just focus on those who have a known mental illness, there will be many who are at risk of suicide that are not attended to. If we put the location of disturbance* in the mental health and not the social arena, we can then make suicide solely the responsibility of mental health services and we can turn away and think no more about it.

### No. 7: we cannot read other people's minds

With time to reflect, the ‘truth’ that we cannot read other people's minds seems undeniable. We don't know what is going on in our own mind much of the time, as around 90% of its functioning is unconscious. In mental health services, however, there is an underlying omnipotent belief, supported by societal expectations, that we have a special intuition about others’ minds and can predict an action someone else is going to make in the future.

*Case example: J had responded well to treatment and was discharged from mental health services by mutual agreement. Nine months later the treating team were very distressed and shocked to hear of his death. The mental health organisation had an internal inquiry where they found fault in J's treatment and linked it with his death in the serious incident report*.

Sometimes the death occurs shortly after being seen by family, friends or clinicians. Understandably, those in most recent contact can feel that they missed something or were misled. However, mental states change rapidly. The person themselves may not have been feeling suicidal at the time of meeting or may not have been aware of their level of distress, or, as described in the case example below, a loss event may have happened in the intervening period.

*Case example: P died in prison. Prior to his death he had been seen by the in-reach GP service. The prison conducted an inquiry and found the GP service negligent for not referring P to the mental health team. It was only disclosed at the coroner's court that 20 min before his death P had a phone call where he was told he could not see his children again*.

### No. 8: no one is to blame for someone else's death by suicide

*… the dead person is the apparent victim, but the true victim is the one that stays alive, for he or she has to live with what they feel they might have caused … the survivor directs enormous resistances to any change or working through of this state, almost as a memorial to the dead*.

Campbell and Hale^8^

When affected by suicide, the capacity to mentalise*, to think and symbolise*, is lost.^33^ In this non-mentalising state, the only option is to think concretely, and shades of grey cannot be conceptualised. ‘Blame’ is a word that comes out of this non-mentalising state of mind. It implies that there is one aetiological factor, and that we know what this is. In reality this could not be further from the truth. Suicide is multifactorial and complex. ‘Responsibility’ is a mentalising word and allows for many different factors to be involved. We can then think about what our responsibility was, and different narratives can be explored, including the part the deceased has played in their own demise.

## Conclusion

### Suicide prevention

There is good evidence for some strategies for suicide prevention. Population-based, public-health-led interventions such as reduction in access to means reduce the suicide rate,^34^ and presentations of self-harm to services give an important opportunity for compassionate intervention. Self-harm is the most important known risk factor for suicide, because there is evidence of the breakdown of symbolisation and a failure to keep distress in the psychic realm.^35^ If this has happened before, it is likely to happen again.

In addition, if suicide is an ‘acting out’ of distress, then to help put the feelings into words, to encourage expression of pain with open-hearted curiosity in our personal and therapeutic relationships, will reduce the risk of action. By speaking we also mitigate stigma, give time and space to those who are experiencing a suicidal crisis, allow those who have survived suicide attempts to process their action, and provide compassionate care to those bereaved.

### The nature of suicide

Suicide has been fundamental to human existence throughout time and across cultures. Concern about suicide cannot just be focused on those working in mental health, giving them the impossible task of predicting the unpredictable and controlling the uncontrollable. Our focus on risk assessment and prediction of suicide in mental health settings sets us a goal that we can only fail in.^36^ It also distracts us from our primary task, which is therapeutic engagement to improve quality of life. In research we conducted, around two-thirds of psychiatrists and other clinicians felt it was their job to predict suicide.^3,4^ Our fantasy that we can do this, and our fear that we can't, becomes a constant preoccupation in our work, distracts us from providing therapeutic care and closes our hearts to those in distress. To approach each patient with anxiety and fear is unlikely to reduce the psychic pain that leads to suicide. To increase our capacity for open-hearted therapeutic engagement will.

If you have had a patient die by suicide, please see this resource on the Royal College of Psychiatrists website: https://www.rcpsych.ac.uk/members/workforce-wellbeing-hub/if-a-patient-dies-by-suicide.

## About the author

Rachel Gibbons is a consultant psychiatrist, psychoanalyst and group analyst. She works independently. She is chair of the Working Group on the Effect of Suicide and Homicide on Clinicians, co-chair of the Patient Safety Group and vice-chair of the Psychotherapy Faculty at the Royal College of Psychiatrists, London, UK.
